# Persistent gestational trophoblastic disease: results of MEA (methotrexate, etoposide and dactinomycin) as first-line chemotherapy in high risk disease and EA (etoposide and dactinomycin) as second-line therapy for low risk disease

**DOI:** 10.1054/bjoc.2000.1176

**Published:** 2000-04-03

**Authors:** L S Dobson, P C Lorigan, R E Coleman, B W Hancock

**Affiliations:** Gestational Trophoblastic Disease Centre, Weston Park Hospital, Sheffield, UK

**Keywords:** gestational trophoblastic disease, combination chemotherapy

## Abstract

Persistent gestational trophoblastic disease is potentially fatal, but the majority of patients are cured with chemotherapy. Any developments in treatment are therefore being directed towards maintaining efficacy and reducing toxicity. We evaluated efficacy and toxicity of methotrexate, etoposide and dactinomycin (MEA) as first-line therapy for high risk disease and etoposide and dactinomycin (EA) as second-line therapy for methotrexate-refractory low risk disease in a retrospective analysis of 73 patients (38 MEA, 35 EA) treated since 1986 at a supra-regional centre. The median follow-up period was 5.5 years and the median number of cycles received was 7. The overall complete response rate was 85% (97% for EA, 75% for MEA). Of eight patients who failed to respond, four have since died and four were cured with platinum-based chemotherapy. Alopecia was universal. Grade II or worse nausea, emesis, or stomatitis was observed in 29%, 30% and 37% respectively. Fifty-one per cent experienced grade II/III anaemia, 8% grade II or higher thrombocytopenia and 64% grade III or IV neutropenia; in six cases this was complicated by sepsis. Fifty-four per cent of patients went on to have a normal pregnancy. No patient has developed a second malignancy. In conclusion, the MEA and EA chemotherapy regimens for persistent trophoblastic disease are very well tolerated, do not appear to affect future fertility and are associated with excellent, sustained complete response rates. © 2000 Cancer Research Campaign


*British Journal of Cancer* (2000) **82**(9), 1547–1552
© 2000 Cancer Research Campaign
DOI: 10.1054/ bjoc.2000.1176, available online at http://www.idealibrary.com on IDEAL®

### Persistent gestational trophoblastic disease: results of MEA (methotrexate, etoposide and dactinomycin) as first-line chemotherapy in high risk disease and EA (etoposide and dactinomycin) as second-line therapy for low risk disease

**LS Dobson, PC Lorigan, RE Coleman and BW Hancock**

Gestational Trophoblastic Disease Centre, Weston Park Hospital, Sheffield, UK

**Summary** Persistent gestational trophoblastic disease is potentially fatal, but the majority of patients are cured with chemotherapy. Any developments in treatment are therefore being directed towards maintaining efficacy and reducing toxicity. We evaluated efficacy and toxicity of methotrexate, etoposide and dactinomycin (MEA) as first-line therapy for high risk disease and etoposide and dactinomycin (EA) as second-line therapy for methotrexate-refractory low risk disease in a retrospective analysis of 73 patients (38 MEA, 35 EA) treated since 1986 at a supra-regional centre. The median follow-up period was 5.5 years and the median number of cycles received was 7. The overall complete response rate was 85% (97% for EA, 75% for MEA). Of eight patients who failed to respond, four have since died and four were cured with platinum-based chemotherapy. Alopecia was universal. Grade II or worse nausea, emesis, or stomatitis was observed in 29%, 30% and 37% respectively. Fifty-one per cent experienced grade II/III anaemia, 8% grade II or higher thrombocytopenia and 64% grade III or IV neutropenia; in six cases this was complicated by sepsis. Fifty-four per cent of patients went on to have a normal pregnancy. No patient has developed a second malignancy. In conclusion, the MEA and EA chemotherapy regimens for persistent trophoblastic disease are very well tolerated, do not appear to affect future fertility and are associated with excellent, sustained complete response rates. © 2000 Cancer Research Campaign

**Keywords:** gestational trophoblastic disease; combination chemotherapy

Trophoblastic disease represents a spectrum of conditions ranging from hydatidiform mole to choriocarcinoma. Persistent trophoblastic disease (PTD) may follow either a molar pregnancy (complete or partial hydatidiform mole) or a non-molar event, for example an ectopic or normal pregnancy. In the UK, all patients diagnosed with a molar pregnancy are followed up with regular β human chorionic gonadotrophin (hCG) urinalysis. In the majority of patients the trophoblastic disease remits following one or more uterine evacuations and there is no need for systemic therapy. In those where trophoblastic disease persists, the criteria for chemotherapy are as follows: an hCG greater than 20 000 iu/L after one or two uterine evacuations; a static or rising hCG level after one or two uterine evacuations; persistent uterine haemorrhage with a raised hCG level; pulmonary metastases with static or rising hCG levels; metastases in liver, brain or gastrointestinal tract; and a histological diagnosis of choriocarcinoma.

In this paper we report our experience using chemotherapy regimens that have been formulated in Sheffield, are more conservative than many equivalent therapies, appear well tolerated and are seemingly very effective. Intravenous (i.v.) methotrexate, etoposide and dactinomycin (MEA) chemotherapy is administered as first-line chemotherapy for patients with high risk disease and in the form of etoposide and dactinomycin (EA) to those patients requiring salvage chemotherapy following unsuccessful low dose intramuscular (i.m.) methotrexate chemotherapy for low risk disease. Toxicity, both acute and long-term, response to therapy and overall survival have been evaluated.

*Received 20 September 1999*
*Revised 14 January 2000*
*Accepted 25 January 2000*

*Correspondence to:* BW Hancock, Yorkshire Cancer Research Section of Clinical Oncology, Weston Park Hospital, Sheffield, UK

### PATIENTS AND METHODS

Sheffield is one of three supra-regional screening centres (and one of two treatment centres) in the UK for the management of gestational trophoblastic disease. All patients are initially managed locally by a gynaecologist and by uterine evacuation(s). Patients with PTD are admitted to this unit for assessment; a history is taken and a physical examination (including a pelvic examination) performed. Serum βhCG level, chest X-ray, computerized tomographic (CT) scan of the chest and an ultrasound scan of the abdomen and pelvis are evaluated and a risk score assigned using the Sheffield modification of the Charing Cross system (Table 1) (Bagshawe et al, 1976; Sheridan et al, 1993). Approximately 5% of all patients registered received chemotherapy, with 80% being deemed to have low risk disease and 20% high risk disease. The patients continued on chemotherapy until 8 weeks after achieving a biochemical complete response (CR), that is normal serum βhCG levels.

A retrospective analysis of the case notes of all women treated with chemotherapy since 1986 was carried out. All women who received first-line high risk chemotherapy with MEA and those who initially received low risk chemotherapy with i.m.

**Table 1** Charing Cross prognostic scoring system

| Variable | 0 | 1 | 2 | 6 |
|---|---|---|---|---|
| Age (years) | < 39 | > 39 | | |
| Antecedent pregnancy (AP) | Mole | Abortion or unknown | Term | |
| Interval from AP to treatment (months) | < 4 | 4–6 | 7–12 | > 12 |
| βhCG (IU $l^{-1}$) | $10^3$–$10^4$ | < $10^3$ | $10^4$–$10^5$ | > $10^5$ |
| ABO blood group (female × male) | | A × O<br>O × A<br>O or A × unknown | B × A or O<br>AB × A or O | |
| Number of metastases | Nil | 1–4 | 4–8 | > 8 |
| Site of metastases | Lungs, vagina | Spleen, kidneys | Gastrointestinal tract, liver | Brain |
| Largest tumour mass | < 3 cm | 3–5 cm | > 5 cm | |
| Previous chemotherapy | Nil | | Single drug | Two or more drugs |

**Table 2** The MEA and EA chemotherapy regimens

MEA chemotherapy
- Methotrexate 300 mg $m^{-2}$ i.v.
- Folinic acid rescue, 15 mg 6-hourly to commence 24 h after chemotherapy, eight doses, the first four being given intravenously.
- 7-day break
- Etoposide 100 mg $m^{-3}$ $day^{-1}$ i.v. for 3 days
- Dactinomycin, 0.5 mg $day^{-1}$ i.v. for 3 days.
- 7-day break and repeat from methotrexate

EA chemotherapy
- Etoposide 100 mg $m^{-2}$ $day^{-1}$ i.v. for 3 days
- Dactinomycin 0.5 mg $day^{-1}$ i.v. for 3 days
- 7-day break and repeat

These schedules are continued for 8 weeks after complete remission (normal βHCG levels).

**Table 3** Clinical features of patients treated

| | | MEA chemotherapy (38 patients) | EA chemotherapy (35 patients) |
|---|---|---|---|
| Age (years) | < 39 | 32 (84%) | 32 (91%) |
| | > 39 | 6 (16%) | 3 (9%) |
| Antecedent pregnancy (AP) | Mole | 21 (55%) | 33 (94%) |
| | Term | 12 (32%) | 1 (3%) |
| | Other | 5 (13%) | 1 (3%) |
| Time from AP (months) | < 4 | 24 (63%) | 21 (60%) |
| | 4–6 | 7 (18%) | 12 (34%) |
| | 7–12 | 3 (8%) | 1 (3%) |
| | > 12 | 4 (11%) | 1 (3%) |
| Presence of liver and/or brain metastases | | 5 (13%) | 0 (0%) |

methotrexate who, because of toxicity or refractory disease, subsequently received second-line EA chemotherapy were identified.

The following information was collected: reason for chemotherapy; number of treatment cycles received; response to treatment; clinical, haematological and biochemical toxicity experienced (graded by the common toxicity criteria); and evidence of fertility.

Patients with high risk disease (score ≥ 8) received intravenous MEA chemotherapy (Table 2) with i.v. dexamethasone and granisetron, for anti-emesis. Patients with central nervous system (CNS) involvement (either a cerebral metastasis on CT scan or a cerebrospinal fluid to venous blood hCG ratio of greater than 1:60) received intrathecal methotrexate (12.5 mg) and a higher dose of i.v. methotrexate (1 g $m^{-2}$). Patients with low risk disease (score ≤ 7) received i.m. methotrexate, 50 mg on alternate days for four doses with folinic acid rescue. There was a 7-day interval between courses. Patients with unacceptable toxicity or disease refractory to first-line therapy (that is static or rising serum βhCG) received salvage therapy with EA chemotherapy (Table 2).

### RESULTS

### Patient characteristics

Between 1986 and 1997, 4677 patients were registered with gestational trophoblastic disease and 224 (4.8%) required treatment. Seventy-seven patients received either MEA (40 patients) or EA (37 patients). Complete data were available for 73 patients (38 received first-line MEA chemotherapy, and 35 patients second-line EA chemotherapy). Their clinical features are summarized in Table 3. A total of 194 patients were scored as having low risk disease and received i.m. methotrexate with folinic acid rescue. Thirty-five (18%) of these received second-line EA chemotherapy. Three were converted to EA therapy as a result of unacceptable methotrexate toxicity (two with abdominal pain, one with pleurisy) and 32 patents (16.5%) had methotrexate refractory disease. A median of six courses of low dose methotrexate (range 2–10 courses) was given prior to conversion to second-line therapy. Thirty-two patients were evaluable to determine the efficacy of EA chemotherapy in methotrexate-resistant disease

 © 2000 Cancer Research Campaign

**Table 4** Effects of treatment

| | MEA chemotherapy | EA chemotherapy |
|---|---|---|
| No. patients in each group | 38 | 35 |
| Complete response rate | 75% | 97% |
| No. patients alive and disease free | 34 | 34 |
| Toxicity | | |
| Nausea – grade II/III | 32% | 26% |
| Vomiting – grade II/III/IV | 26% | 28% |
| Stomatitis – grade II/III | 37% | 37% |
| Alopecia – grade III | 100% | 100% |
| Cough | 26% | 17% |
| Conjunctivitis – grade I/II | 76% | 20% |
| Anaemia – grade II/III | 50% | 51% |
| Thrombocytopenia | 8% | 9% |
| Neutropenic sepsis | 10.5% | 6% |
| Patients requiring G-CSF support | 18% | 17% |
| Patients requiring blood transfusion | 39% | 46% |
| Patients requiring treatment delay | 3% | 6% |
| Patients requiring i.v. antibiotics | 13% | 14% |
| Patients requiring oral antibiotics | 42% | 26% |
| Abnormal liver enzymes – grade II/III | 24% | 0% |
| Patients with a subsequent normal pregnancy | 48% | 60% |

and 35 were evaluable for toxicity. All patients receiving first-line MEA were evaluable for response and toxicity.

### Response to treatment (Table 4)

A total of 744 cycles of treatment were prescribed, 487 MEA and 257 EA. The median number of cycles of both EA and MEA received was seven (range 1.5–12 cycles). The CR rate for all patients was 85%. In patients receiving second-line EA therapy for methotrexate refractory low risk disease, the CR rate was 97%. The one patient who failed EA chemotherapy is currently alive but with active disease; histology at presentation was a complete mole and on relapse was a classical choriocarcinoma.

The CR rate for patients receiving MEA as first-line therapy was 75%. Two patients received intrathecal methotrexate and high dose methotrexate (1 g $m^{-2}$) for CNS disease, one also had a cerebral metastasis resected. The latter was one of eight patients with refractory disease who required second line platinum-containing treatment; of these four were cured. Thirty-four patients (89%) treated initially with MEA are alive and disease-free. Four have died as a result of their disease; all were over the age of 40, in three cases the histology was not that of classical choriocarcinoma (two had placental site trophoblastic tumours) and one patient had CNS involvement.

The median number of years of follow-up was 5.5 years (range 10 months to 11.5 years).

### Toxicity (Table 4)

### *Haematological*

EA chemotherapy was associated with grade III/IV neutropenia in 20 patients (57%), affecting 41 courses (16% total number of courses). In two patients the neutropenia was complicated by sepsis. Five patients (14%) required i.v. antibiotics during their chemotherapy for non-neutropenic episodes; in each patient only one course was affected. Nine patients (26%) required oral antibiotics.

MEA chemotherapy caused grade III/IV neutropenia in 26 patients (68%), affecting 54 courses (11% of total number of courses). In four patients this was complicated by sepsis. Five patients (13%) required i.v. antibiotics, with 16 (42%) receiving oral antibiotics during their chemotherapy.

Two patients (8%) receiving EA exhibited grade III anaemia during treatment, only three courses were affected. Sixteen patients (46%) exhibited grade II anaemia and 16 patients were transfused a total of 50 units of blood. MEA was associated with grade III anaemia in one patient (3%) and grade II in 18 (47%); 15 patients required a blood transfusion, receiving a total of 58 units.

One patients receiving EA exhibited grade IV thrombocytopenia after one course. The patient required 10 units of platelets. One patient on MEA also exhibited grade IV thrombocytopenia as a result of disseminated intravascular coagulopathy secondary to infection.

Forty-two courses (5.8%) of treatment received by 13 patients (seven MEA, six EA) were supported with granulocyte-colony stimulating factor. In all 13 cases this was due to previous grade III or IV neutropenia; four patients also exhibited documented sepsis, and in eight patients treatment had been compromised by delay. In one patient the neutropenia was not complicated by either infection or delay in subsequent treatment course. A treatment delay of over 14 days was observed in three patients (two patients on EA), and in each case this was a result of neutropenic sepsis.

### *Non-haematological toxicity*

Nine patients (26%) receiving EA exhibited grade II or III nausea. Grade III or IV vomiting was observed in three cases (8%). Twelve patients (32%) on MEA had grade II or III nausea. Grade III vomiting only was seen in one patient. All patients exhibited temporary grade III alopecia.

Grade II or III stomatitis was experienced in 13 patients (37%) on EA and 14 patients (36%) on MEA. This affected 20 courses (9%) and 25 courses (6%) respectively. Eight patients (23%) receiving EA and 16 (42%) receiving MEA experienced a skin

© 2000 Cancer Research Campaign

**Table 5** Comparison of the toxicity associated with EMA/CO and MEA/EA

| No. of cycles (%) | | | | | | |
|---|---|---|---|---|---|---|
| **Haematological** | **EMA/CO Grade III** | **MEA Grade III** | **EA Grade III** | **EMA/CO Grade IV** | **MEA Grade IV** | **EA Grade IV** |
| Neutropenia | 21 | 8 | 11 | 12 | 3 | 5 |
| Anaemia | 4 | 0.2 | 1 | 1 | 0 | 0 |
| Thrombocytopenia | 2 | 0 | 0 | 1 | 0.2 | 0.3 |
| **Non-haematological** | **EMA/CO Grade 1** | **MEA Grade I** | **EA Grade I** | **EMA/CO Grade II** | **MEA Grade II** | **EA Grade II** |
| Nausea | 13 | 8 | 13 | 10 | 3 | 7 |
| Stomatitis | 8 | 8 | 6 | 7 | 4 | 7 |
| Dermatitis | 5 | 4 | 3 | 0.8 | 2 | 2 |
| Pleuric pain | 4 | 0 | 0 | 0 | 0 | 0 |
| Diarrhoea | 3 | 0 | 0 | 2 | 0 | 0 |
| Conjunctivitis | 18 | 4 | 2 | 0 | 2 | 0.4 |
| Neuropathy | 0.8 | 0 | 0 | 0.8 | 0 | 0 |

rash which was self-limiting at some stage during their treatment. Six patients (17%) on EA and ten (26%) on MEA experienced a cough. Formal pulmonary toxicity with spirometry was not assessed. Seven patients (20%) receiving EA and 29 (76%) receiving MEA exhibited grade I or II conjunctivitis.

Twenty-seven courses of therapy were modified (12 courses of EA and 15 of MEA). Nine patients (24%) who received MEA chemotherapy experienced grade II/III abnormalities in hepatic transaminases. Methotrexate was omitted in three high risk patients, with two receiving EA and one continuing with dactinomycin only. The latter patient was on a drug rehabilitation programme and her hCG levels had returned to normal. No patient in the EA group exhibited grade II or higher alteration in hepatic transaminases. Impairment of renal function was not observed.

### *Long-term effects*

Thirty patients (55%) became pregnant after chemotherapy; this included two ectopic pregnancies and one miscarriage. Eighteen patients have not had a further pregnancy and no information is available on their current fertility status. Fertility was not ascertained in 12 patients as it was too soon after chemotherapy. Six women had a hysterectomy and three were using regular contraception. Three patients, two who had EA, were attempting to become pregnant. There have been no documented cases of second malignancy.

### DISCUSSION

The criteria for initiating chemotherapy for PTD vary from centre to centre. In the USA a standard recommendation is that if βhCG levels increase or plateau over a period of 3 or more consecutive weeks, immediate work-up and treatment for PTD are indicated (Goldstein and Berkowitz, 1995). At the other end of the spectrum, UK clinicians advocate a more expectant policy and in low risk cases are prepared to observe patients for up to 6 months with serial βhCG estimations. Five per cent of patients registered at this centre receive chemotherapy (Doreen et al, 1993; Sheridan et al, 1993; Hancock et al, 1997) compared with 7–8% at Charing Cross, UK (Newlands 1997) and up to 20% in the USA (Kennedy et al, 1995).

### The Charing Cross experience

Patients are scored using the Charing Cross System (Table 1) and are classified as being low risk (0–5), intermediate risk (6–9) or high risk (> 9). Patients with low risk disease receive i.m. methotrexate with folinic acid rescue. Twenty-five per cent require alternative chemotherapy, which is virtually always successful (Bagshawe et al, 1989; Newlands et al, 1997). In our study population, where low risk was defined by a score of 0–7, alternative chemotherapy (in the form of EA) was required in 18% of these patients; the complete response rate with EA was 97%.

High risk patients (score > 9) are treated with EMA/CO (etoposide [100 mg $m^{-2}$ i.v. on days 1 and 2], methotrexate [300 mg $m^{-2}$ i.v. on day 1, followed at 24 h by folinic acid 15 mg 12-hourly hourly $\times$ 4], dactinomycin [0.5 mg i.v. on days 1 and 2]/cyclophosphamide [600 mg $m^{-2}$ i.v. on day 8] vincristine [1.4 mg $m^{-2}$ i.v. on day 8]); in addition patients with pulmonary metastases are given CNS prophylaxis with intrathecal methotrexate (Bower et al, 1997). Overall survival was 85% when EMA/CO it was given to 148 patients between 1979 and 1989 (Newlands et al, 1991). There were two categories of treatment failure. In 76 patients who received EMA/CO as first-line therapy, ten of the 14 cases progressed on therapy having had extensive disease at diagnosis. The overall response rate was 82%. This was similar to our overall response rate of 80%, however it is worth noting that in our study, patients received MEA with a risk score of 8 or higher, compared with above 9 for EMA/CO at Charing Cross; CNS prophylaxis was not given (Gillespie et al, 1999). In 72 patients who had received prior chemotherapy either at Charing Cross or at another hospital, the survival was better, with 64 patients being cured (89%). In the eight patients who failed EMA/CO, the principal cause of death was drug resistance. Salvage chemotherapy for high risk patients consisted of cisplatin with etoposide alternating with the EMA schedule and achieved an 82% response rate in this setting (Newlands et al, 1991).

### The American experience

In the USA, patients are usually staged using either the International Federation of Gynaecology and Obstetrics (FIGO) anatomical staging system (FIGO, 1992) or Hammond's clinical

 © 2000 Cancer Research Campaign classification for gestational trophoblastic disease (Hammond et al, 1973). Whilst direct comparisons of these classifications with the Charing Cross or WHO (World Health Organization) scoring systems is not possible there is reasonable equivalence (Welsh et al, 1999); thus similar groups of patients are selected for multiagent chemotherapy.

The New England Trophoblastic Disease Centre protocol for management of FIGO stage I disease is based upon the desire to preserve fertility. In patients who wish to preserve fertility, single-agent chemotherapy is given with methotrexate or dactinomycin. A complete remission was seen in 385 (93%) of 414 patients with stage I disease (Berkowitz and Goldstein, 1997). This is higher than response rates for first-line therapy in low risk disease reported from the UK but probably reflects the fact that in general, more patients are treated, 20% compared to 5–10%. It is possible that this represents treatment of some patients that would otherwise have been cured by curettage alone and in whom unnecessary exposure to cytotoxic therapy could have been avoided. The remaining 29 resistant patients (7%) later achieved remission with either combination chemotherapy or surgical intervention. If a patient no longer wishes to preserve fertility, hysterectomy (with adjuvant single-agent chemotherapy) may be performed as primary treatment. Patients with stage II and III disease are assessed for prognostic factors using the WHO scoring system (WHO, 1987). The prognostic factors are similar to those used in the Charing Cross system but have a different weighting attached. In general, low risk patients are treated with primary single-agent chemotherapy and high risk patients are managed with primary combination chemotherapy. A review of four reports shows 87% remission rate with single-agent chemotherapy and an eventual remission rate of 98.6% (DuBreschter et al, 1987; Dubuc-Lissor et al, 1989; Ayhan et al, 1992; Soper et al, 1994*a*). Low risk patients who fail first-line chemotherapy with methotrexate in the New England Trophoblastic Center receive MAC (originally methotrexate, 0.3 mg $kg^{-1}$ i.m., dactinomycin 8–10 μg $kg^{-1}$ i.v. and chlorambucil 0.2 mg $kg^{-1}$ orally or cyclophosphamide 250 mg i.v.: each is given daily for 5 days and the cycle is repeated every 14 to 21 days) or EMA/CO chemotherapy. Similar to our experience, all patients with methotrexate-resistant disease achieved a complete response (Berkowitz and Goldstein, 1997). Management of stage IV disease includes primary intensive chemotherapy with sequential methotrexate, dactinomycin and cyclophosphamide, and selective use of radiotherapy and surgery (Lurain, 1994). Using this combination, approximately 80% of patients will achieve a remission – similar to our experience with MEA.

### Comparisons between the different chemotherapy regimens

Unlike MEA and EMA/CO, MAC does not contain etoposide. The dose of etoposide in EMA/CO is 66% of that in MEA (200 mg $m^{-2}$ vs 300 mg $m^{-2}$). Etoposide has been reported to be associated with an increased risk of secondary tumours including leukaemia, breast cancer, colon cancer and melanoma. At present, there have been no reported second malignancies in our patients treated with etoposide for gestational trophoblastic disease, but follow-up is short and we have recently reduced the time period for ‘consolidation’ chemotherapy from 8 to 6 weeks.

There have been many studies reported on the use of combination chemotherapy in high risk disease and in patients failing single-agent therapy (Hammond et al, 1973; Newlands et al, 1986; Quinn et al, 1994; Soper et al, 1994*b*). In internationally reported series MAC produced complete responses in 66% (113/170) of patients when given as first line therapy (Lurain, 1994). Early reports of therapy with EMA/CO were more encouraging with 93% (103/111) of patients responding completely to this regimen as first-line and 78% (80/102) as second-line treatment (Lurain, 1994). MEA as first-line and EA as second-line therapy appear to be equally effective – 85% and 97% respectively. However, as emphasized previously, different centres use different criteria to determine high risk disease so these results, though roughly equivalent, may not be truly comparable.

Table 4 summarizes haematological and non-haematological toxicity associated with EMA/CO (Newlands et al, 1991) and MEA,EA. More courses of EMA/CO were associated with grade III/IV anaemia, neutropenia and thrombocytopenia (5%, 33% and 3%) compared to MEA,EA (0.5%, 13%, 0.3%).

Grade I/II nausea was seen with both treatments (23% EMA/CO, 15% MEA,EA). The incidence of stomatitis and dermatitis were the same for both treatments; conjunctivitis was more frequent with EMA/CO (18% compared with 5%) and no patients receiving MEA or EA had problems with neuropathy, diarrhoea or pleuritic chest pain.

MAC chemotherapy was associated with significant myelotoxicity after 2–3 treatment cycles. In approximately 6% this was life-threatening and anaemia was a frequent complication (Hammond et al, 1973).

In women treated with EMA/CO, Bower et al (1997) reported the occurrence of second malignancies, most commonly acute myeloid leukaemia, associated with etoposide administration. We have as yet failed to demonstrate the occurrence of second malignancies with MEA and EA, with a median follow-up of 5.5 years (range 10 months to 11.5 years). This may be as a result of a relatively short follow-up time and fewer patients assessed.

We conclude that the regimens (MEA, EA) which we have consistently used since 1986 for high risk and methotrexate-resistant PTD are as efficacious as, and possibly better tolerated than, any other regimen reported to date for these two clinical situations.

### REFERENCES

Ayhan A, Yapar EG, Deren O et al (1992) Remission rates and significance of prognostic factors in gestational trophoblastic tumours. *J Reprod Med* **37**: 461–465

Bagshawe KD (1976) Risk and prognostic factors in trophoblastic neoplasia. *Cancer* **38**: 1373–1385

Bagshawe KD, Dent J, Newlands ES et al (1989) The role of low-dose methotrexate and folinic acid in gestational trophoblastic tumours. *Br J Obstet Gynaecol* **96**: 795–802

Berkowitz RS and Goldstein DP (1997) Presentation and management of persistent gestational trophoblastic disease and gestational trophoblastic tumours in the USA. In: *Gestational Trophoblastic Disease*, 1st edn, Hancock BW, Newlands ES, Berkowitz RS (eds), pp 159–172. Chapman and Hall, London

Bower M, Newlands ES, Holden L et al (1997) EMA/CO for high-risk gestational trophoblastic tumours: results from a cohort of 272 patients. *J Clin Oncol* **15**: 2636–2643

Doreen MS (1993) Gestational trophoblastic diseases: a review of their presentation and management. *Clin Oncol* **5**: 46–56

DuBreschter B, Berkowitz RS, Goldstein DP et al (1987) Metastatic gestational trophoblastic disease: experience at the New England Trophoblastic Disease Centre, 1965–1985. *Obstet Gynaecol* **69**: 390–395

Dubuc-Lissor J, Sweizig S, Schlaerth JB et al (1989) Metastatic gestational trophoblastic disease: a comparison of prognostic classification systems. *Gynaecol Oncol* **45**: 40–45

FIGO Oncology Committee Report (1992) *Int J Gynaecol* **39**: 149–150
Gillespie AM, Siddiqui N, Coleman RE and Hancock BW (1999) Gestational trophoblastic disease: does central nervous system chemoprophylaxis have a role? *Br J Cancer* **79**: 1270–1272
Goldstein DP and Berkowitz RS (1995) Prophylactic chemotherapy of complete molar pregnancy. *Semin Oncol* **22**: 157–160
Hammond CB, Borchert LG, Tyrey L et al (1973) Treatment of metastatic disease: good and poor prognosis. *Am J Obstet Gynaecol* **115**: 451–457
Hancock BW (1997) Difference in management and treatment: a critical appraisal. In: *Gestational Trophoblastic Disease*, 1st edn, Hancock BW, Newlands ES, Berkowitz RS (eds), pp 213–216. Chapman and Hall, London
Kennedy AW (1995) Persistent non-metastatic gestational trophoblastic disease. *Semin Oncol* **22**: 161–165
Lurain JR (1994) High-risk metastatic gestational trophoblastic tumours: current management. *J Reprod Med* **39**: 217–222
Newlands ES (1997) Presentation and management of persistent gestational trophoblastic disease and gestational trophoblastic tumours in the UK. In: *Gestational Trophoblastic Disease*, 1st edn, Hancock BW, Newlands ES, Berkowitz RS (eds), pp 143–156. Chapman and Hall, London
Newlands ES, Bagshawe KD, Begent RHJ et al (1986) Developments in chemotherapy for medium and high risk patients with gestational trophoblastic tumours, 1979–84. *Br J Obstet Gynaecol* **93**: 63–69
Newlands ES, Bagshawe KD, Begent RHJ et al (1991) Results with the EMA/CO (etoposide, methotrexate, actinomycin D, cyclophosphamide, vincristine) regimen in high risk gestational trophoblastic tumours, 1979–1989. *Br J Obstet Gynaecol* **98**: 550–557
Quinn M, Murray J, Friedlander M et al (1994) EMACO in high-risk gestational trophoblastic disease; the Australian experience. *Aust NZ J Obstet Gynaecol* **34**: 90–92
Sheridan E, Hancock BW, Smith SC et al (1993) Gestational trophoblastic disease: experience of the Sheffield (United Kingdom) supra regional screening and treatment service. *Int J Oncol* **3**: 149–155
Soper JT, Clarke-Pearson DL, Berchuck A et al (1994*a*) 5-day methotrexate for women with metastatic gestational trophoblastic disease. *Gynaecol Oncol* **54**: 76–79
Soper JT, Evans AC, Clarke-Pearson DL et al (1994b) Alternating weekly chemotherapy with etoposide-methotrexate-dactinomycin/cyclophosphamide-vincristine for high risk gestational trophoblastic disease. *Obstet Gynaecol* **83**: 113–117
Welsh E, Gillespie AM, Hancock BW (1999) Assessing and staging trophoblastic tumours – a comparison of the various systems. *Br J Cancer* **80**: 110
WHO World Health Organization, Geneva (1987) *Trophoblastic diseases. WHO Tech Rep Ser 692*

*© 2000 Cancer Research Campaign*
